# Myointimoma (angiocentric myofibroblastic tumor) of the glans penis in an adolescent: a case report and review of the literature

**DOI:** 10.1186/s12894-022-01131-3

**Published:** 2022-11-16

**Authors:** Marcel Drlík, Mária Gregová, Josef Sedláček, Radim Kočvara

**Affiliations:** 1 Division of Paediatric Urology, Department of Urology, First Faculty of Medicine, General Teaching Hospital and Charles University First Faculty of Medicine, Prague, Czech Republic; 2 Institute of Pathology, First Faculty of Medicine, Charles University and General University Hospital in Prague, Prague, Czech Republic

**Keywords:** Myointimoma, Penile tumor, Adolescent, Case report

## Abstract

**Background:**

Soft tumors of the penis are extremely rare in childhood and adolescence. Because there are no guidelines, each such case raises embarrassment over the extent and degree of aggressiveness of the diagnostic and therapeutic procedures. Herein, we describe a case of a teenager with a penile myointimoma along with a review of the literature. So far, only 10 cases have been reported in this age group.

**Case presentation:**

The 15-year-old boy presented with a 6-months history of a slowly growing, palpable firm nodule in glans penis. Physical examination revealed a palpable, well circumscribed, firm, whitish painless mass, around 1 cm in diameter within the glans. Ultrasound revealed hypoechogenic, hypoperfused poorly defined area inside the glans. MRI did not confirm any other pathologic mass inside the glans and corpora cavernosa. An excisional biopsy was performed; its immunohistological features indicated myointimoma.

**Discussion and conclusion:**

Given the rarity of this benign entity, the main importance is to distinguish myointioma from more clinically aggressive neoplasms. The key to a correct diagnosis is a careful histological examination, including immunohistochemistry. Local excision is safe and effective treatment modality.

## Background

Myointimoma, also known as angiocentric myofibroblastic tumor, is a rare benign soft tissue neoplasm derived from intimal cells of the vascular spaces of the corpora cavernosa of the penis, histologically characterized by multinodular / plexiform myofibroblastic proliferation within the vascular spaces of cavernous bodies. The term myointimoma was first introduced by Fetsch et al. [1] in 2000 and recognized as a distinctive histological entity in the World Health Organization Classification of the Tumors of the Urinary System and Male Genital Organs in 2016 [2]. So far, only 22 cases have been described in the literature, of which only 10 in children and adolescents. Except of two small series [1, 3] of cases that are based on a retrospective re-evaluation of few decades stored slides of tumors, these are always isolated case reports. Given the rarity of this tumor, the main importance is to distinguish myointimoma from other neoplasm of variable biological behavior. Our aim is to describe an additional case of myointimoma in an adolescent and provide a review of the literature focusing on children and adolescents.

## Case presentation

**Fig. 1:**
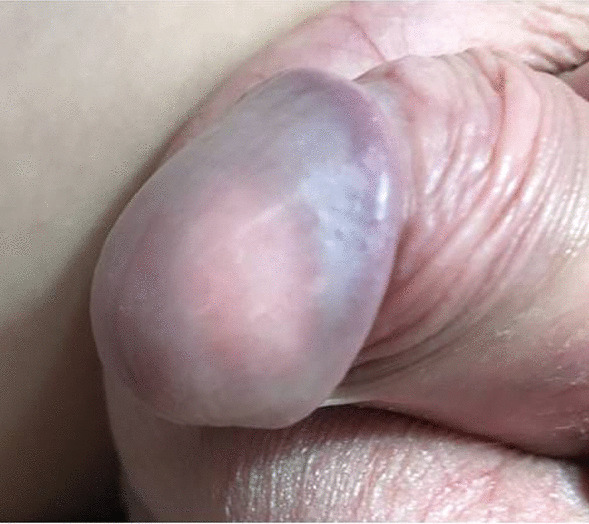
Whitish nodule visible under normal overlying skin

A 15-year-old Caucasian boy presented with a 6-months history of a slowly growing, palpable firm nodule within glans penis. Clinically he was completely asymptomatic and voided freely. The patient did not report any history of trauma, systemic connective tissue diseases or other autoimmune disorders. On physical examination, there was a palpable, well circumscribed, firm, whitish painless mass, around 1 cm in diameter within the glans (Fig. 1). The overlying skin was of a normal structure without signs of inflammation. No palpable inguinal lymphadenopathy was observed. The stage of puberty was Tanner III.

**Fig. 2:**
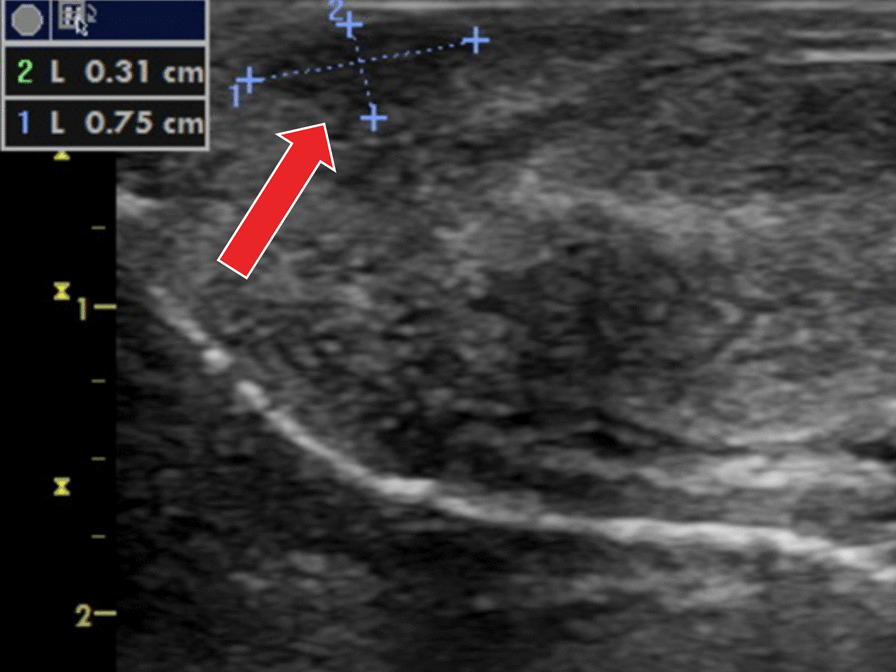
Ultrasound finding—a hypoechogenic, hypoperfused non-well defined area inside the glans (arrow)

**Fig. 3:**
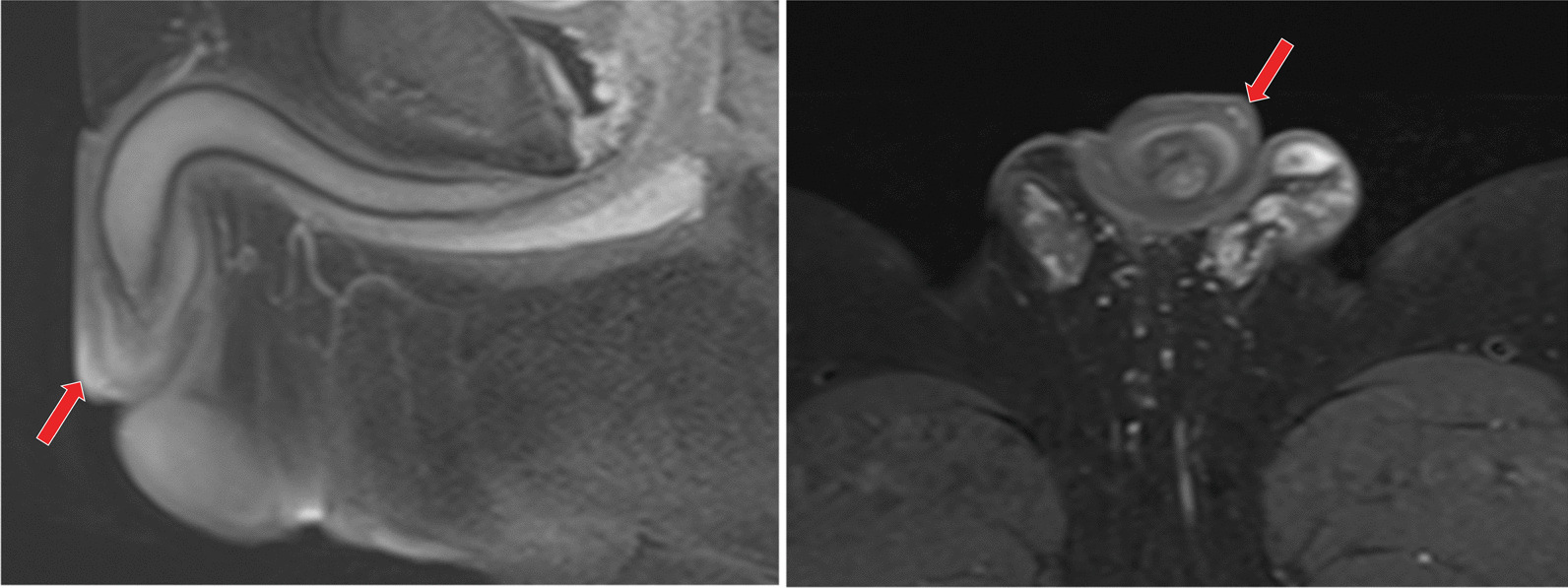
MRI finding—a single hyperintense mass inside glans (arrow), corpora cavernosa are normal, sagittal (**A**) and coronal (**B**) cut

**Fig. 4:**
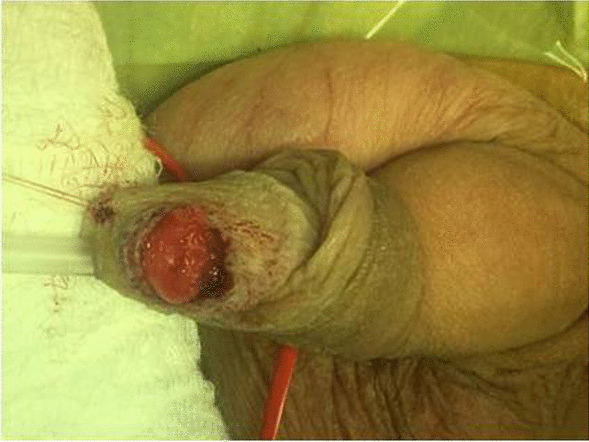
Careful excisional biopsy with special attention to protection of the urethra

**Fig. 5:**
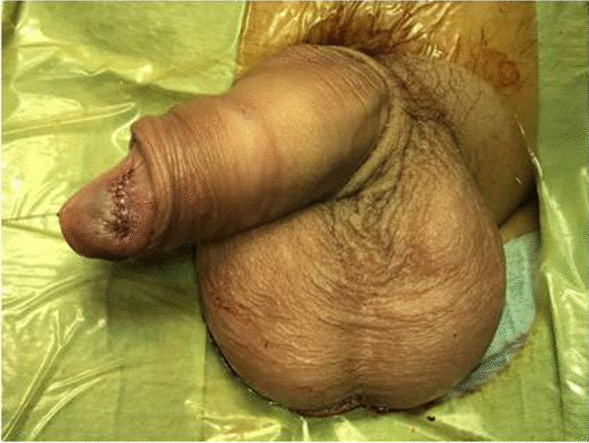
Simple wound closure

As there are no guidelines concerning penile tumors in this age, we adhered to the EAU guidelines for penile cancer in adults and performed penile Doppler Ultrasonography and MRI (Magnetic Resonance Imaging). Ultrasound revealed hypoechogenic, hypoperfused poorly defined area inside the glans (Fig. 2). MRI did not confirm any other pathologic mass inside the glans and corpora cavernosa (Fig. 3). An excisional biopsy under general anesthesia with intra-operative pathological evaluation was decided. The formation was not clearly demarcated from the surrounding glans tissues and reached close to the urethra, without interfering with its wall. The procedure was performed at optical magnification, using magnifying glasses with particular attention to prevent the injury of the neighbouring urethra (Fig. 4). As the intra-operative pathological evaluation showed a benign nature of the tumor, we simply closed the wound and did not proceed with any more extensive surgery (Fig. 5).

**Fig. 6:**
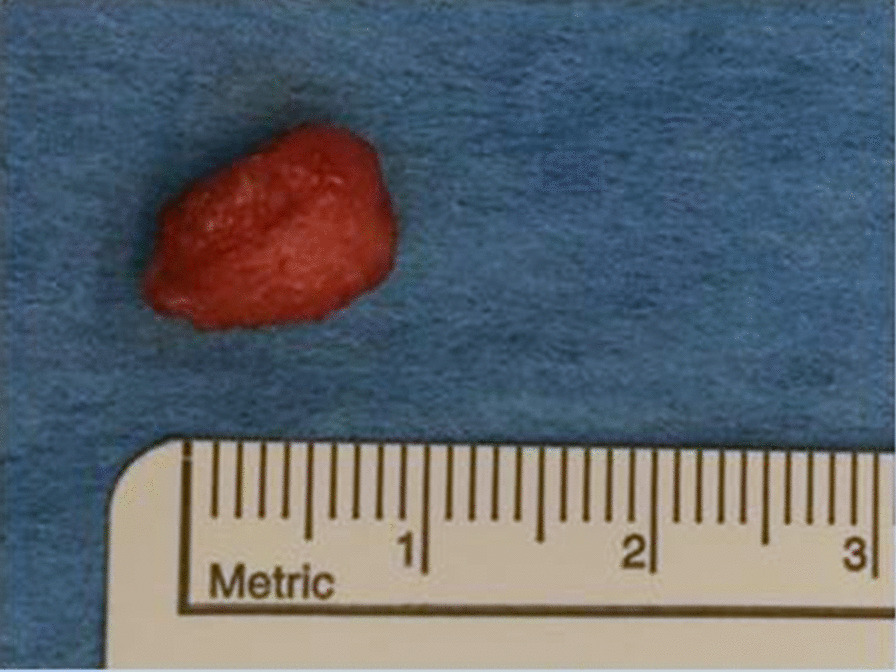
Macroscopic apperance of myointimoma

**Fig. 7:**
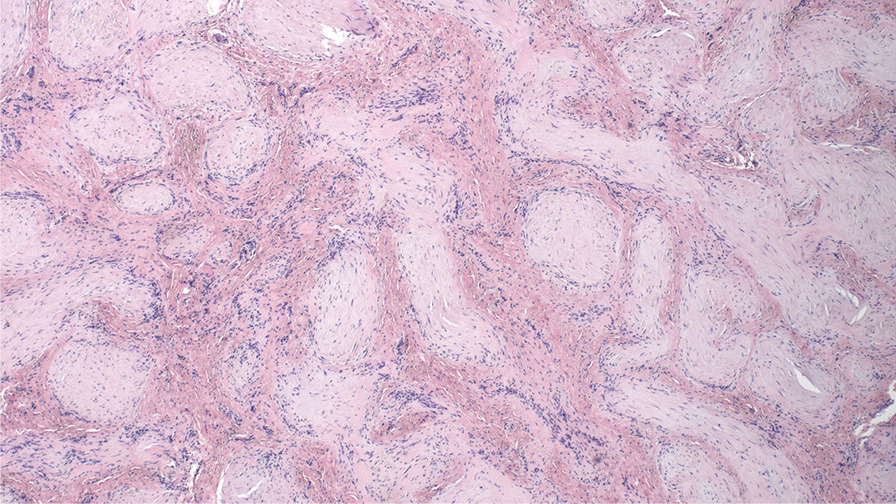
Nodular intravascular proliferation of spindle myofibroblastic cells presenting typical morphology for myointimoma (hematoxylin-eosin, 100x)

**Fig. 8:**
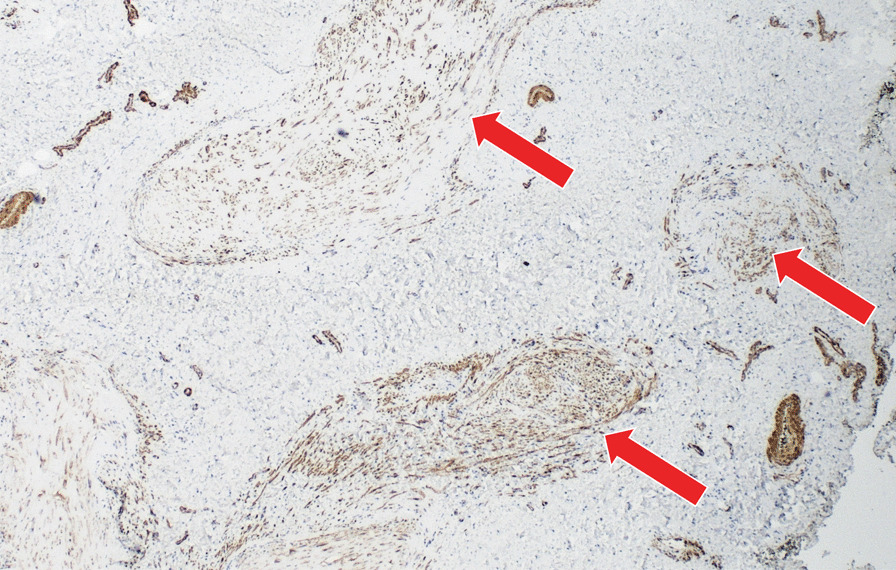
Immunohistochemical expression of alpha-smooth muscle actin (SMA) shows diffuse positivity in intravascular myofibroblastic population (arrow) (200x)

**Fig. 9:**
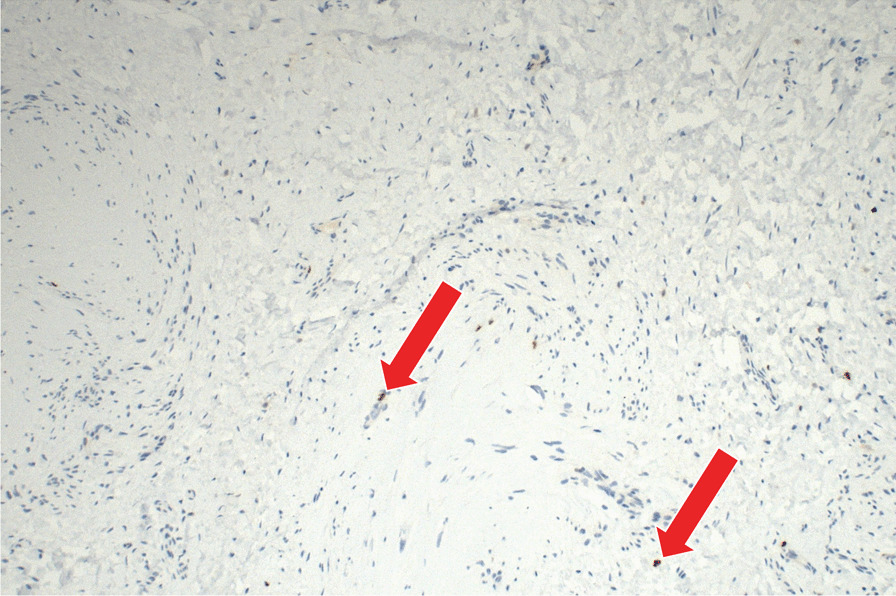
Immunohistochemical expression of Ki-67 (proliferative antigen MIB-1), labelling cells beyond G0 phase of the mitotic cycle, shows expression of sporadic cells (beneath 1%, arrow) (400x)

**Fig. 10:**
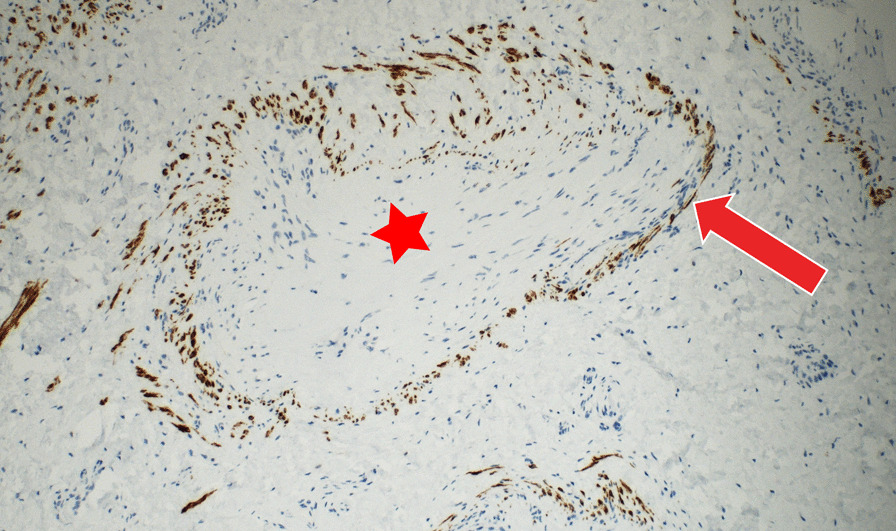
Immunohistochemical expression of desmin showing positivity in the smooth muscle cells of the pre-existing vessel walls (arrow), tumorous myofibroblastic cells are negative (star) (400x)

We obtained a macroscopically pale tissue sample measuring 10 × 8 × 5 mm (Fig. 6). On the cut surface, the lesion was light red in colour and had solid consistency. Subsequent detailed histopathological analysis revealed changes diagnostic for myointimoma - nodular intravascular myofibroblastic proliferation involving multiple cavernous spaces (Fig. 7). At low power magnification, a complex multinodular architecture was seen. At higher magnification the myofibroblasts were uniform, elongated spindle shaped cells with no significant hyperchromasia or pleomorphism, nor any mitotic figures or necrosis. Immunohistochemical staining for alpha-smooth muscle actin (αSMA) was positive intralesionaly (Fig. 8), proliferative activity (Ki-67) was low (beneath 1%) (Fig. 9). Immunostaining for desmin was negative in myofibroblasts, while positive in the pre-existing vessel wall only (Fig. 10). No reactivity was seen for other performed immunohistochemical markers (S100 protein, CD34 and ERG) (Tables 1, 2). Microphotographs were taken with Olympus BX41 microscope and processed by QuickPHOTO Software.

**Table 1:** Cases of myointimoma in children and adolescents – clinicopathologic features

Reference	Age (years)	Location	Size (cm)	Treatment	Recurrence
Fetch et al.	2	Glans	0.5	NR	NR
	2	Glans, near meatus	1	Excisional biopsy	No
	4	Glans near corona	0.7	Punch biopsy	Regression
Mc Kenney et al.	12	Glans, right side	0.4	Excisional biopsy	No
	4	Glans right side	0.7	Excisional biopsy	No
	9	Glans left side	0.5	Excisional biopsy	No
	15	Glans left side	1.8	Excisional biopsy	No
	9	Glans	1	Excisional biopsy	No
Turner et al.	14	Glans, right side	1	Incisional biopsy	Stable anatomy
Tannnirvedi et al.	11	Glans, left side	1	Excisional biopsy	No

*NR* not reported

**Table 2:** Summary of assessed immunohistochemical markers

Immunohistochemical antibodies	Usual positive staining	Result in myointimoma
Alpha-smooth muscle actin (SMA)	Smooth muscle cells, myofibroblasts, myoepithelial cell, osteoblasts, chondrocytes, pericytes, and others	Positive
Ki-67	Marker of proliferative aktivity	Low (beneath 1%)
Desmin	Myoblasts, myofibroblasts (variable), myometrium, smooth muscle cells, and others	Negative in tumor, positive in the pre-existing vessel wall
S100 protein	Marker of cells of neuroectodermal histogenesis (neurons, schwann cells, melanocytes, glial cells), myoepithelial cells, adipocytes, Langerhans cells, dendritic cells, interdigitating dendritic cells, chondrocytes and notochordal cells	Negative
CD34	Endothelium of blood vessels, hematopoietic progenitor cells, and others	Negative
ERG	Blood vessel and lymphatic endothelial cells, immature myeloid cells	Negative

**Fig. 11:**
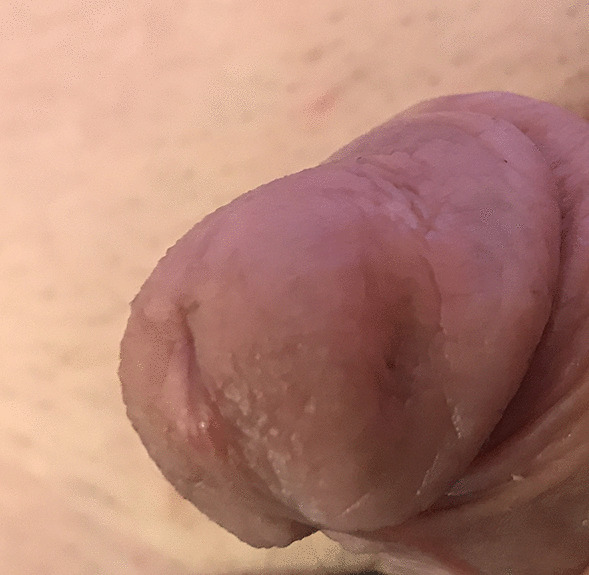
Favourable cosmetic outcome 3 years later

Due to the benign nature of the lesion, we did not perform staging for distant metastases and simply performed an outpatient follow-up. Three years after the excision, there is no local recurrence, no urethral stricture and a cosmetic appearance is good (Fig. 11).

## Discussion and conclusion

This case report refers an additional case to the 10 previous reported cases of myointimoma in children and adolescents [1, 3–5], (Table 1). In our case, like in all previously reported cases in adolescents and adults [1, 6–10], the myointimoma affects uniquely the glans penis. Likewise, no reported case was associated with pain, dysuria or signs of lower urinary tract obstruction. In our case, the patient reported a relatively fast-growing mass. The history of initial rapid growth is common in the literature; later however, the formation may remain stable over time. Monsalves [7] described a case of myointimoma that remained unchanged 10 months after an incomplete excision. Fetsch [1] described the same experience with a 6-month stable residual mass in a patient after an incisional biopsy. In one case, complete regression of myointimoma at 10-years follow-up was described [1]. Local aggressive growth or distant metastases were never reported.

There are currently no guidelines describing the extent of imaging in adolescents with penile tumors. The existing literature does not deal with the scope of imaging; both existing series [1, 3] of cases are based on a retrospective re-evaluation of stored hematoxylin and eosin-stained slides of penile tumors over the last few decades only. Therefore, we adhered to EAU guidelines for penile cancer in the adults and performed penile Doppler Ultrasonography and MRI to exclude corporal invasion. The examinations confirmed the solid nature of the tumor, excluded cystic lesion and multiple involvement of cavernosal tissue. In a case of penile tumor in adolescents, the main concern was to exclude clinically aggressive conditions, thus an excisional biopsy was decided. Since the boy was confirmed to have benign findings on histopathological examination and had clinically normal findings on the inguinal nodes, we did not perform staging (abdominal, pelvic and thoracic CT).

The diagnosis of myointimoma and its differential diagnosis based on morphology only may be confusing. There are several types of mesenchymal tumors with plexiform or nodular structure. Immunohistochemistry is a key to exact diagnosis. Myointimomas always express alpha-smooth muscle actin (αSMA). Desmin may be absent or show only focal reactivity. There is no reactivity for S-100 protein, CD31, CD34, ERG, epithelial membrane antigen (EMA) or neuron specific enolase (NSE). The plexiform growth pattern can be found in plexiform histiocytic tumor (PFHT) [11]. Unlike myointimoma, it contains a mixture of two components: a differentiated spindle fibroblastic/myofibroblastic cells and a round histiocytic cell component containing multinucleated giant cells (osteoclast-like giant cells). Immunohistochemically, the histiocytes and multinucleated giant cell express CD68, whereas the spindle cells express αSMA. PFHT may recur and has a low risk of metastases (lymph node, lung). A plexiform or nodular growth pattern can we see in some nerve sheet tumors such as plexiform schwannoma [12] or neurofibroma. Immunohistochemical expression for S-100 protein is then helpful in differential diagnosis.

The myointimoma structure may resemble myofibroma, a more common tumor in children. In contrast, it does not exhibit the exclusive intravascular growth; the growth is rather concentric around the small vessels. The tumor is composed of oval or spindle myoid cells [13]. Myopericytomas characterized by a distinctive biphasic growth pattern, with central hypercellular zone composed of spindle tumor cells, hyalinization and myoid cell nodules visible towards the periphery of the tumor. In contrast to myofibroma, intravascular growth is more common in myopericytoma, but it does not indicate a malignant neoplatic process [14].

Epithelioid hemangioma and hemangioendothelioma can be distinguished from myointimoma by immunostaining as the endothelial nature of the lesional cells can be confirmed by CD31, CD34 and ERG positivity. Another structurally similar pathology is a late phase of intravascular fasciitis (intravascular nodular fasciitis). Histologically, intralesional inflammatory cells between spindle myofibroblast cells, mucoid pools, a less compact stroma with more eosinophilic hyalinization, and obvious mitotic figures were observed [1]. Intravascular spindle cells lesion such as intravascular leiomyoma or leiomyomatosis can be easily distinguished by immunohistochemistry, with αSMA, desmin and h-caldesmon antibodies, which are typically strongly positive. Last but not least, the possibility of sarcoma with angioinvasive spread must be ruled out at the histological examination.

Both clinician and pathologist must be aware of this rare benign entity. The key to a correct diagnosis is a careful histological examination of the specimen, including immunohistochemistry. Local excision is safe and effective treatment modality.

## Data Availability

All data generated or analysed during this study are included in this published article.

## Abbreviations

MRI: magntic resonance imaging
EAU: European Association of Urology
αSMA: Alpha-smooth muscle actin
EMA: Epithelial membrane antigen
NSE: Neuron specific enolase
PFHT: Plexiform histiocytic tumor
